# Concentration, Health Risk, and Hydrological Forcing of Heavy Metals in Surface Water Following Water-Sediment Regulation of the Xiaolangdi Dam in the Yellow River

**DOI:** 10.3390/ijerph19095713

**Published:** 2022-05-07

**Authors:** Qinghe Zhao, Shengyan Ding, Zihan Geng, Xunling Lu, Zhendong Hong, Yi Liu, Jinhai Yu

**Affiliations:** Key Laboratory of Geospatial Technology for the Middle and Lower Yellow River Regions of the Ministry of Education, College of Geography and Environmental Science, Henan University, Kaifeng 475004, China; zhaoqinghe@henu.edu.cn (Q.Z.); 104753190167@henu.edu.cn (Z.G.); luxunling@henu.edu.cn (X.L.); zdhong@henu.edu.cn (Z.H.); liuyi@henu.edu.cn (Y.L.); yjh666@henu.edu.cn (J.Y.)

**Keywords:** water-sediment regulation, trapping effect, hydrological forcing, heavy metals, health risk

## Abstract

Water and sediment regulation aimed at aquatic ecosystems and preserving reservoir capacity to minimize the negative consequences of dams can fundamentally change the distribution of heavy metals (HMs) in the reservoir and downstream reaches. However, the effects of water and sediment regulation on variation in HMs are still poorly understood. In this study, the variations in concentration, contamination, human health risk, potential sources, and influencing factors of the metalloid As and HMs (Cr, Hg, Ni, Pb, and Zn) in surface water in the reservoir and the downstream reach of the Xiaolangdi Dam (XLD) following the operation of the water-sediment regulation scheme (WSRS) were determined. These results indicate that HM concentrations in the two post-WSRS seasons were much lower than the water quality standards, but were significantly increased over time due to the trapping effects of the XLD (*p* < 0.05, except for Zn). However, As concentration in the reservoir was significantly lower than that observed in downstream reaches, likely due to anthropogenic input from agricultural activities. Meanwhile, HM concentrations varied with distance to the dam, which displayed a distinct accumulation closer to the dam in the post-WSRS II season. The contamination of HMs, the carcinogenic risk of exposure to As, and the noncarcinogenic risks associated with exposure to Hg, Ni, Pb, and Zn via the direct ingestion pathway of drinking water were all within acceptable levels following the WSRS, but increased over time. The carcinogenic risk of Cr in the post-WSRS II season was at an unacceptably high level, particularly at sites near the dam. Hydrological characteristics (water level and flow rate) were the dominant factors in determining the distribution of HMs. These results can provide new insight for a better understanding of the variations in HMs following the water and sediment regulation practices, and guide future management in regulating the trapping effects of dams.

## 1. Introduction

The presence of dams on rivers not only alters hydrological processes but also impedes the flow of essential materials, including water, nutrients, metallic elements, and sediments, leading to enhanced material transformation and accumulation via retention, sedimentation, adsorption, and primary productivity in dammed reservoirs [1,2]. Changes in these essential materials can lead to marked environmental consequences. For example, the reduced intensity and frequency of extreme runoff events could affect the downstream hydraulics and physical habitat required by various aquatic organisms [3,4], while the accumulated metallic elements in reservoirs may create permanent environmental pressure on aquatic organisms [5]. Meanwhile, deposited sediments upstream of the dam could significantly decrease reservoir storage capacity [6], while reduced sediments downstream of the dam could lead to channel incision, bank collapse, and loss of morphology and habitat diversity and connectivity [7,8]. In addition to these environmental consequences, the trapping effects of dams also have the potential to threaten the safety of the dams themselves as well as those living downstream, which may even trigger serious regional tensions [9].

Considering the environmental and social consequences related to the trapping effects of dams, combined with the increasing impacts of climate change, there is growing interest in finding strategies for sustainable management of dammed rivers [2,10]. A variety of solutions have therefore been developed to mitigate the material accumulation upstream of the dam and the material loss downstream of the dam. For example, the mechanisms of energy availability (light and temperature), nutrient input, and the presence of metal-oxide minerals have been implemented to govern the extent of nutrient elimination induced by dams [1,2]. Strategies such as flood flushing, sediment bypassing, and artificial replenishment have been applied to mitigate sediments reduction downstream of the dam [8]. Sluicing operations have been implemented by the Water Framework Directive of the European Community to reestablish sediment continuity and preserve the water storage function of reservoirs [11]. All of these practices focus on regulating reservoir and downstream conditions by manipulating dam operations and have proven to be effective practices in mitigating the trapping effects of dams [12,13]. However, despite the notable effectiveness of these practices in regulating water and sediments, there is a current lack of understanding of how contaminants vary during and after these practices, particularly regarding contaminant dynamics and water quality in reservoirs and the downstream reaches.

During such practices, large quantities of sediments and both dissolved and particulate contaminants would be discharged to the downstream reaches in a short period of time (about one week, depending on the size and silting situation of the reservoir), thus causing a potentially high level of contamination and a decrease in water quality for downstream aquatic environments [11,12]. Evidence suggests that sediments act as a sink for organic and inorganic contaminants due to their high sorption capacity [5,13]. Therefore, the resuspension and redeposition of sediments could inevitably lead to speciation, transport, and bioavailability of contaminants in the downstream reaches [14,15,16], particularly for metallic elements, which are prone to being released from sediment matrices into the water column and becoming bioavailable when facing changes in fluvial environments [11,17,18]. In this regard, sediments in turn act as a source of metallic elements. These metallic elements, particularly the heavy metals (HMs), in aquatic ecosystems are of major concern due to their abundant, persistent bioaccumulation, and toxicity [19,20]. Evidence has suggested that HMs can a pose permanent risk to aquatic biota and humans even at a low concentration [5,21,22]. Therefore, effective river management requires promoting the mechanistic understanding of how these water and sediment regulation practices affect the biogeochemical and physical transformation and accumulation of essential materials [2], particularly the HMs in water. However, the distribution and accumulation of HMs in surface water during and after these water and sediment regulation practices are not well understood.

The lower Yellow River in China is famous for its substantial sedimentation, as 4 × 10^8^ kg per year of suspended sediments derived from the Loess Plateau have been deposited on the riverbed over the last century [23]. To address the issues of channel siltation and water shortages, as well as to control floods in the lower Yellow River, the Xiaolangdi Dam (XLD), located at the exit of the last canyon in the middle reaches of the Yellow River, began construction in September 1991 and started operation in October 1999. To reduce the sediment retention and maintain the capacity of the XLD Reservoir, the water-sediment regulation scheme (WSRS) has been carried out by the Yellow River Conservancy Commission (YRCC) of China since 2002 [24]. Generally, the WSRS lasts for 10–20 days; however, almost half of the total annual sediments accompanied by large amounts of terrestrial materials including nutrients and metallic elements are discharged into the sea during this short period [23,24,25,26]. More than 57% of the annual quantity of particulate HMs was discharged into the ocean during the 2009 WSRS [20], while approximately 30%, 42–54%, and 49–60% of the total annual HM flux were transported to the ocean during the WSRS in 2013 [27], 2015 [25], and 2018 [21], respectively. The dramatic changes in the transportation of HMs have likely driven the growing interest in the distribution and accumulation of HMs following the WSRS. While previously mentioned studies have explored the impacts of the WSRS on the flux of HMs, they were based on HM data observed at the Lijin gauge station, which is located about 740 km downstream from the XLD. However, few studies have investigated the distribution and accumulating process of HMs in surface water following the WSRS. To better understand this process, the spatial distribution of HMs (considering the analogous toxicity of the metalloid As to heavy metals, the term HMs in the present study includes As for convenience of description) from the XLD reservoir to the downstream reach, temporal accumulation of HMs in different seasons after the WSRS, and the factors influencing the spatial distribution and temporal accumulation of HMs in water require further investigation.

Therefore, the main objectives of this study are: (1) to analyze spatial–temporal variations in the concentrations of As Cr, Hg, Ni, Pb, and Zn in surface water of the XLD Reservoir and downstream reach following the 2018 WSRS; (2) to assess the contamination degree and health risk level of HMs in surface water; (3) to determine the potential sources of HMs in surface water of different seasons based on multiple statistical analyses; and (4) to reveal the factors influencing the concentrations of HMs in water. The investigation of these objectives has potential implications for future management in regulating the trapping effects of dams.

## 2. Materials and Methods

### 2.1. Study Area

**Figure 1:**
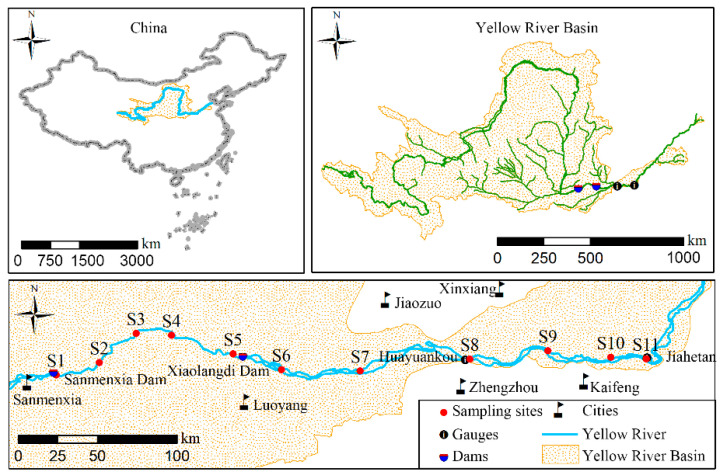
Location of the study area and sampling sites in the Xiaolangdi Reservoir (S1–S5) and the downstream reach (S6–S11) in the middle and lower reaches of the Yellow River.

This work was conducted in the XLD Reservoir and its downstream reach from the outlet to Jihetan hydrological station (Figure 1). The Xiaolangdi Dam (XLD) is the second-largest dam in China. It is the last large dam on the mainstream of the Yellow River with a controlled drainage area of 6.94 × 10^5^ km^2^ and a reservoir storage capacity of 126.5 × 10^8^ m^3^. It controls 91.5% of the total water discharge and 98% of the total sediment discharge of the Yellow River [23]. The XLD primarily serves to reduce siltation, control floods, alleviate water shortages, and generate power for the middle and lower reaches of the Yellow River [20,28]. The XLD Reservoir area is characterized by a temperate continental monsoon climate, with an average annual temperature of 12.4–14.3 °C, average annual precipitation of 616 mm, and average annual humidity of 62%. The downstream reach of the XLD Reservoir passes through the Huang-Huai-Hai Plain with channel gradient ranging from 0.1 to 0.5 m km^−^^1^ [23,29]. Meanwhile, the downstream reach of the XLD Reservoir is famously referred to as a “suspended river” with its riverbed 7–13 m higher than the surrounding landscape and a large area of riparian zones bounded by levees [24,30]. Since the implementation of the WSRS in 2002, drastic alterations in the hydrodynamic conditions of the reservoir area and the downstream reach have occurred [31], which has fundamentally altered the resuspension and redeposition of HMs in water, as well as the interaction and exchange between the riverbed and the water column.

### 2.2. Sample Collection and Analysis

The 2018 WSRS was implemented in July, from the 3rd to the 30th. Water samples were collected from 11 transverse sections in the reservoir and downstream reach in October 2018 in the post-WSRS I season and in April 2019 in the post-WSRS II season (Figure 1 and Table S1). In each season, surface water at a depth of 0.5 m was sampled from 11 transverse sections located in the reservoir area (S1–S5) and the downstream reach (S6–S11) as shown in Figure 1 and Table S1. Each transverse section comprised three sampling sites, specifically, a right, middle, and left site. At each site, three parallel surface water samples were collected by a hydrophore, acidified with HNO_3_ (pH < 2), and stored in polyethylene bottles. The hydrophore and bottles were precleaned with HCl solution (pH = 2) and were then rinsed five times using water to be sampled. All water samples were placed in a car-carried refrigerator and were transported to the Laboratory of Geospatial Technology for the Middle and Lower Yellow River Regions at Henan University. Samples were then stored at 4 °C for later analysis. At each site, pH, dissolved oxygen (DO), and electrical conductivity (EC) of surface water were measured using an SX736 multi-probe. In the laboratory, the concentrations of Cr, Ni, Pb, and Zn were measured via the inductively coupled plasma mass spectroscopy (ICP-MS, Thermo Fisher, Waltham, MA, USA). The concentrations of As and Hg in water samples were measured via atomic fluorescence spectrophotometry (DB51/T836–2008). The precision and accuracy of HM concentrations were assessed using blanks, duplicate samples, and certified standard reference materials for water samples (GBW08607), which were analyzed with groups of 20 samples. The relative standard deviation of HM concentrations among triplicate samples was ±5%. The average recovery of HMs spiked with the standards were in the range of 90% and 110%. These results were acceptable and consistent with certified values, which were expressed as the mean concentration of triplicate samples. The water level and flow rate data at the inlet (Sanmenxia), reservoir (Xiaolangdi Reservoir), outlet (Xiaolangdi), and downstream reaches (Huayuankou, and Jiahetan) gauge stations during the two sampling periods were obtained from the YRCC (http://www.yrcc.gov.cn/ (accessed on 1 September 2021)).

### 2.3. Contamination Evaluation for Heavy Metals

Prior to evaluating the contamination level of HMs, we briefly reviewed the traditionally used geochemical indexing approaches, such as the contamination degree (C_d_) developed by Backman et al. [32], heavy metal evaluation index (HEI) by Edet and Offiong [33], heavy metal contamination index (HCI) by Rajkumar et al. [34], heavy metal pollution index (HPI) by Mohan et al. [35], and modified heavy metal pollution index (m-HPI) by Chaturvedi et al. [36]. We found that C_d_ assumes that the analytical value lower than the upper permissible limit should not pose any environmental hazard; the HEI is easy to calculate but lacks the prescribed scale for assessment purposes; the HPI is widely used but the prescribed scale of 100 is too large to make assessments when the concentration of a particular HM is markedly high; the HCI is similar to HEI, but it classifies the critical value with 5 water classes between 0 to 100, based on mean deviation and percent deviation at each sampling site; and the m-HPI, along with its various modifications, express the pollution status of HMs for any water sample as a paired positive index (PI) and negative index (NI) based on the highest desirable and maximum permissible concentrations, which aims to address the various shortcomings of the existing indexing systems [37]. However, there is currently a lack of further classification when the observed HM concentration is lower than the maximum permissible concentrations, with the logic that the water samples should be classified as excellent when PI equals zero [36]. In addition to the above limitations, the evaluation results of these indices are not always consistent as shown in previous studies [34,36,38]. Therefore, it may become quite confusing when multiple indices are used to assess contamination levels of HMs in water samples. Therefore, we ultimately selected the HCI proposed by [34] to analyze the spatial–temporal variations in the contamination level of HMs following the WSRS. However, we also assessed the HEI described by Edet and Offiong [33] along with two modifications of m-HPI offered by Chaturvedi et al. [36,37] and Sahoo and Swain [39], as shown in the Supplementary Table S2. The calculation of HCI followed that of Rajkumar et al. [34] can be found below.

(1) $$HCI=\sum_{i=1}^{n}MI_{i}$$

(2) $$MI_{i}=W_{i}\times q_{i}$$

(3) $$q_{i}=C_{i}/S_{i}\times 100$$

(4) $$W_{i}=Aw_{i}/\sum_{i=1}^{n}Aw_{i}$$

where *MI_i_* is the sub-index of the *i*th HM, *W_i_* is the unit weight of the *i*th HM, *q_i_* is the rating based on concentrations of the *i*th HM, *C_i_* is the concentrations of the *i*th HM, *S_i_* is the permissible limit of the *i*th HM, *Aw_i_* is the assigned weight of the *i*th HM, and *n* is the number of HMs. The permissible limit and relative weights of individual HMs for HCI can be found in Supplementary Table S3. The scales for contamination levels of HMs in this study referred to the three classes proposed by Edet and Offiong [33] to better characterize moderate levels HMs contamination when all values are less than the critical value of 100 [40,41,42]: low (HCI values < 15), medium (HCI values within 15–30), and high (HCI values > 30).

### 2.4. Human Health Risk Assessment

In this study, the human health risk assessment guide proposed by the US Environmental Protection Agency (EPA), which has been described elsewhere [22,40,41,42,43,44,45], was used to assess human health risk exposure to HMs. Generally, human exposure to heavy metals occurs through several pathways, including direct ingestion, dermal adsorption, and inhalation [46,47,48,49,50,51]. However, ingestion is known to be the most common pathway for HMs in water [40,41,42,43,44]. Meanwhile, human health risks regarding exposure to a specific HM can be classified as either carcinogenic or noncarcinogenic risks [40,44]. For this study, the non-carcinogenic and carcinogenic risk for specific HMs through the direct ingestion route of drinking water was determined for both adults and children [22]. The noncarcinogenic health risk of individual HMs (Hg, Ni, Pb, and Zn) was calculated with Equation (5):

(5) $$R_{i}^{nc}=\left[ADD/\left(RfD_{i}\times 70\right)\right]\times 10^{-6}$$

where $R_{i}^{nc}$ refers to the noncarcinogenic health risk of the *i*th HM, *RfD_i_* refers to the reference dose of the *i*th HM offered by the USEPA [52], 70 is the average human lifespan (years), and ADD is the average daily dose (mg kg^−1^ d^−1^) calculated using Equation (6):

(6) $$ADD=\frac{C\times IR\times EF\times ED}{BW\times AT}$$

where *C* refers to the concentration of the target HM; *IR* refers to the intake rate (kg d^−1^ or L d^−1^), which is 2.2 for adults and 1.0 for children up to 7 years old; *EF* represents the exposure frequency (365 d per year); *ED* is the exposure duration (years) and is equal to 30 years; *BW* is the body weight (kg), which is 64.3 for adult and 22.9 for children of Henan Province; *AT* is the average exposure time (days); and AT is the average exposure time (days).

The carcinogenic health risk of individual HM (As and Cr) was calculated with Equation (7):

(7) $$R_{i}^{c}=ADD\times SF_{i}/70$$

where $R_{i}^{c}$ refers to the carcinogenic health risk of the *i*th HM and *SF_i_* refers to cancer slope factor in units of mg kg^−1^ day^−1^ offered by the USEPA [52]. *ADD* for the carcinogenic health risk is the same as that of the noncarcinogenic health risk; however, *AT* is 30 years for carcinogens. Following the USEPA guidance, risk values lower than 1 × 10^−6^ are not considered to pose significant carcinogenic health effects, values higher than 1 × 10^−4^ signify a high cancer risk to humans, and values falling within the range of 1 × 10^−6^ to 1 × 10^−4^ are generally considered acceptable [42,45].

### 2.5. Statistical Analysis

To determine how HM concentrations varied between the two seasons following the WSRS and between the XLD Reservoir and its downstream reach, a two-way analysis of variance (ANOVA) with a statistical significance level of 5% (*p* < 0.05) was performed using SPSS v.22.0 software (IBM, Chicago, IL, USA). To analyze the potential sources of HMs in surface water in different seasons, the positive matrix factorization (PMF) and hierarchical cluster analysis (HCA) were performed using EPA PMF 5.0 program and OriginPro2021 software (version 9.8.5, OriginLab Corporation, Northampton, MA, USA), respectively. To determine the correlation between HM concentrations and hydrological and physicochemical characteristics, redundancy analysis (RDA) was performed using OriginPro2021 software. Prior to RDA, the mean concentration of each individual HM at S1 and S2 was paired with the hydrological and physicochemical characteristics observed at the inlet station (Sanmenxia). In the same way, S3–S5 were paired with the reservoir station (Xiaolangdi Reservoir), S6 and S7 with the outlet station (Xiaolangdi), S8 and S9 with the downstream station at Huayuankou (HYK), and S10 and S11 with the downstream station at Jiahetan (JHT).

## 3. Results and Discussion

### 3.1. Spatial–Temporal Variation in Concentration of Heavy Metals in Surface Water

Table 1 displays the estimated descriptive statistics for HM concentrations in surface water in the reservoir and the downstream reach of the Xiaolangdi Dam in the post-WSRS I (former) and post-WSRS II (latter) seasons. Generally, average concentrations of As, Cr, Hg, Ni, Pb, and Zn were lower than the criteria for drinking water proposed by the Ministry of Health of China [46] and the World Health Organization (WHO) [47], with values ranging from 0.36 to 5.51, 0.87 to 4.40, 0.02 to 0.09, 1.81 to 9.43, 1.10 to 5.38, and 25.61 to 40.81 μg/L, respectively. This result suggests that HMs may not pose a major challenge to the water quality of the XLD Reservoir and its downstream reach. The highest level of As and Zn was observed in the downstream reach in the latter season, while relatively low concentrations of As and Zn were observed in the reservoir during the same season. Temporally, concentrations of As in the former season were significantly lower (*p* < 0.05) than that in the latter season. Spatially, the concentration of As in the downstream reach was significantly higher (*p* < 0.05) than that in the reservoir. In contrast, Zn did not exhibit any significant difference temporally or spatially, though the temporal and spatial differences were obvious. The higher concentrations of Hg, Ni, and Pb were found in the reservoir, while the low values were measured in the downstream reach. However, there was no significant difference between the reservoir and the downstream reach, except for Ni, which displayed significant accumulation in the reservoir in comparison with the downstream reach. A higher concentration of Cr was found in the downstream reach in the former season, while it was measured in the reservoir in the later season.

**Figure 2:**
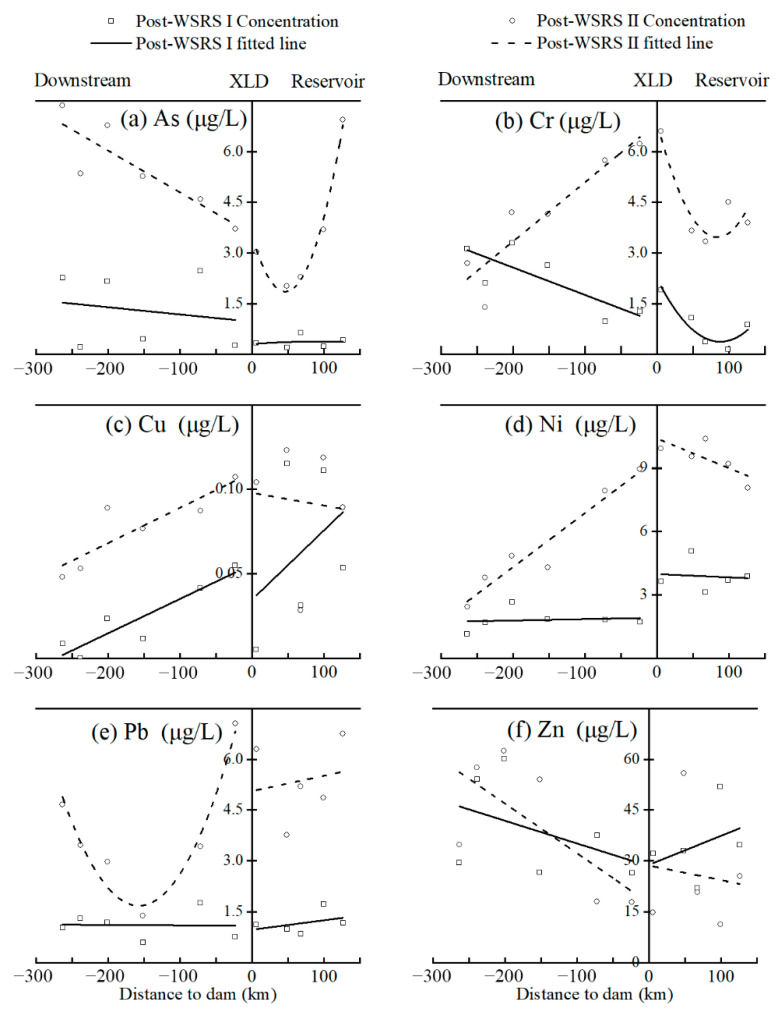
Concentrations of As (**a**), Cr (**b**), Cu (**c**), Ni (**d**), Pb (**e**), and Zn (**f**) in relation to distance above and below the Xiaolangdi Dam in the post-WSRS I and post-WSRS II seasons.

Additionally, HM concentrations in surface water varied with distance to the dam (Figure 2). Specifically, in the reservoir area, all HMs in the former season did not show distinct accumulation near the dam, except for Cr, which showed a distinct accumulation closer to the dam in the latter season. In the downstream reach, the concentration of As, Cr, and Zn generally increased, Cu decreased, and Pb and Ni exhibited no apparent change with distance to the dam in the former season; the concentration of As and Zn generally increased, Cr, Cu, and Ni decreased, and then Pb decreased first and then increased with distance to the dam in the latter season.

The results of this research are in agreement with the previous reports stating that concentrations of the selected HMs were lower than their corresponding limits for drinking water in both the mid-WSRS season [12] and the post-WSRS seasons [48]. However, inconsistent results were observed regarding the spatial difference between the reservoir and the downstream reach. Previous studies reported no significant difference regarding HM concentrations (As, Cr, Pb, Cu, Ni, and Zn) between the reservoir and the downstream reach [12], while significant differences were observed for As and Ni in the present study. This inconsistency may ascribe to the regulating effects of the WSRS and the trapping effects of the XLD. During the WSRS, the artificially released sediment from the Xiaolangdi Reservoir may adsorb HMs in the surface water, which could reduce the concentrations and regional differences in dissolved HMs along the downstream reach [5,17,20,25,49]. In contrast, during the post-WSRS seasons, the trapping effects of the XLD could enlarge these regional differences between the reservoir and the downstream reach, which has been suggested by several previous studies [14,22,41]. This may also explain the accumulation of HMs near the dam in the reservoir area [5]. Additionally, besides Zn, concentrations of the other five HMs in the latter season were significantly higher than those in the former season, regardless of whether they were in the reservoir area or the downstream reach. This is likely attributed to the unsteady flow conditions at the initial stage after the WSRS, which disequilibrates the adsorption–desorption processes between the particulate and dissolved HMs, resulting in more dissolved HMs being adsorbed on the surface of suspended sediments [1,2]. Consequently, HM concentrations were relatively low in the post-WSRS I season due to the sorption effects of suspended sediments, which is consistent with the result observed during the WSRS within the study area [12]. In contrast, the steady-state flow condition after completion of storing water in the reservoir was favorable for HMs to reach the adsorption–desorption equilibrium state in the latter season, as it is characterized by slow flow rate and significant sedimentation and desorption of suspended sediments [3,4,22]. Therefore, the suspended and deposited sediments desorbed more dissolved HMs into the water, which lead to higher HM concentrations in the later season [12,13]. Meanwhile, the trapping effect of the dam is also known to cause the accumulation of HMs over time [5,8]. Furthermore, As and Cr concentrations, which showed quadratic variations in the reservoir in the later season, should be attributed to the releasing effect of the Sanmenxia Reservoir and the trapping effect of the XLD Reservoir, which led to high values of As and Cr occurring downstream near the Sanmenxia Dam and in the XLD reservoir before the dam. Pb concentrations in the downstream also showed quadratic variations in the later season, which should be attributed to the exogenous input from agricultural practices further downstream the XLD [31]. Additionally, the HM concentrations obtained in this study were comparable to those of the other water bodies in China, for example, the Three Gorges Reservoir [14,22], Taihu Lake [50], and Wen-Rui Tang River [42]. However, HM concentrations in the present study were lower than those in Mangla Lake, Rawal Lake, and Simly Lake in Pakistan [43] as well as the average values of HMs in surface water of India, South Africa, Iran, and the USA as reviewed by Kumar et al. [51].

### 3.2. Assessing the Contamination and Human Health Risk of Heavy Metals in Surface Water

**Figure 3:**
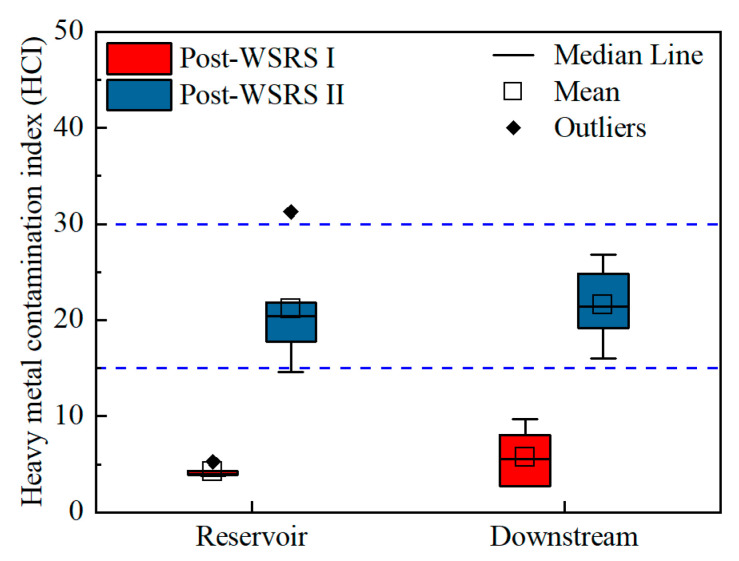
Contamination of heavy metals in surface water of the Xioalangdi Reservoir and the downstream reach following the WSRS.

The calculated heavy metal contamination index (HCI) values in the reservoir area ranged from 3.76 to 5.26 during the post-WSRS I season (former) and from 14.59 to 31.27 during the post-WSRS II (latter) season. In the downstream reach, the HCI value ranged from 2.73 to 9.71 during the former season and from 15.99 to 26.78 during the latter season (Figure 3), confirming that all HCI values were less than the critical value of 100 for drinking water [33]. However, the contamination of HMs in surface water following the WSRS increased over time. Meanwhile, the contamination of HMs increased slightly along the flow direction. To better characterize the moderate levels of HMs contamination, the HCI values below 100 were classified into three classes [42]. In the former season, all HCI values were lower than 15, suggesting a low HM contamination level at the former stage following the WSRS. In contrast, a medium HMs contamination level was measured at the latter stage following the WSRS. Among the HMs, As and Pb contributed the most to HCI, accounting for 43.05% and 42.18% in the former season and 29.98% and 48.27% in the latter season, respectively. HM contaminations were most severe around monitoring site S1 (31.27) in the latter season. This observation may be mainly attributed to the release of accumulated HMs trapped by the Sanmenxia Dam, as S1 is located downstream of the dam outlet. It is well documented that As and Pb contamination has been a global environmental issue due to their toxicity and persistence [41,47]. Therefore, contamination by As and Pb should be of great concern in the study area.

In general, water in the XLD Reservoir and the downstream reach were not distinctly contaminated by HMs, though the HCI values were observed to increase over time. These findings highlight the efficiency of the WSRS in mitigating the trapping effects of the XLD, which greatly alleviates the material siltation in the reservoir area and the material loss in the downstream reach [1,2,8,10,11]. Indeed, in comparison with measurements taken prior to the operation of the WSRS in 1999–2001 (311 million tons), the annual sediment retention for 2002–2013 (262 million tons) was much lower following the operation of the WSRS [49].

**Figure 4:**
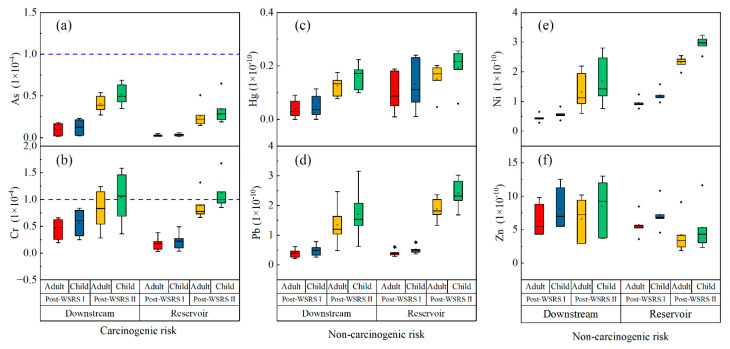
Carcinogenic risk of As (**a**) and Cr (**b**) and Non-carcinogenic risk of Hg (**c**), Pb (**d**), Ni (**e**), and Zn (**f**) in surface water of the Xiaolangdi Reservoir and the downstream reach in the post-WSRS I and II seasons. The dashed line shows the unacceptable limit for the carcinogenic risks. The non-carcinogenic risks were all far below the unacceptable limit. The diamond marker stands for outliers.

Figure 4 presents the carcinogenic and non-carcinogenic risks of HMs via the direct ingestion pathway of drinking water. In the reservoir area, the carcinogenic risk from exposure to As in surface water for adults and children in the former season was 0.14–0.47 × 10^−5^ and 0.18–0.60 × 10^−5^, respectively, reaching 1.48–5.09 × 10^−5^ and 1.88–6.49 × 10^−5^ in the latter season (Figure 4a). The carcinogenic risk associated with exposure to As for adults and children in the downstream reach was 0.16–1.66 × 10^−5^ and 0.21–2.30 × 10^−5^ in the former season, respectively, reaching 2.72–5.39 × 10^−5^ and 3.48–6.88 × 10^−5^ in the latter season. According to the criteria established by the USEPA [52], the values of carcinogenic risk for As were all within the acceptable range of 1 × 10^−6^ to 1 × 10^−4^, suggesting that there may be inconspicuous adverse effects on the health of residents through the ingestion pathway [42,45]. However, the carcinogenic risk of As via the direct ingestion pathway of drinking water increased over time (Figure 4b). The carcinogenic risk of Cr intake through the ingestion pathway for adults and children in the former season also were at acceptable levels, with mean values of 1.75 × 10^−5^ and 2.23 × 10^−5^ in the reservoir area, and mean values of 4.47 × 10^−5^ and 5.70 × 10^−5^ in the downstream reach, respectively. However, they were at unacceptably high levels in the latter season, as the carcinogenic risk of Cr ranged from 0.67 × 10^−4^ to 1.32 × 10^−4^ for adults (1/5 of sites exceeded the critical level) and 0.85 × 10^−4^ to 1.68 × 10^−4^ for children (2/5 of sites exceeded the critical level) in the reservoir area, and then ranged from 0.28 × 10^−4^ to 1.24 × 10^−4^ for adults (2/5 of sites exceeded the critical level) and 0.36 × 10^−4^ to 1.59 × 10^−4^ for children (4/5 of sites exceeded the critical level) in the downstream reach. Meanwhile, the highest values of carcinogenic risk for Cr were found in the site surrounding the dam in both the reservoir (S5) and the downstream reach (S6). The above findings imply that residents living near the dam are exposed to adverse carcinogenic effects from Cr through the ingestion pathway of drinking water [40,44], particularly children. In addition, although the highest carcinogenic risks of Cr were observed near the dam and then decreased away from the dam in the reservoir area and as the river flows down, the health risk caused by Cr may still pose a significant issue as residents living in the area surrounding the reservoir and the downstream reach may be exposed to Cr through other pathways, including direct and indirect [41,45]. Assessing the carcinogenic risk of As and Cr based on the direct ingestion pathway of drinking water alone, likely underestimates the real health risk to residents via exposure.

Like the carcinogenic risk assessment, the noncarcinogenic risks to adults and children were calculated. The noncarcinogenic risks posed by the four HMs were relatively low in this study, suggesting that no apparent noncarcinogenic health risks were observed study area following the WSRS [40]. Specifically, noncarcinogenic risks of Hg (Figure 4c), Pb (Figure 4d), Ni (Figure 4e), and Zn (Figure 4f) ranged from 0 to 2.01 × 10^−11^, 0.21 to 2.47 × 10^−10^, 0.28 to 2.54 × 10^−10^, and 1.84 to 10.18 × 10^−10^ for adults, and 0 to 2.56 × 10^−11^, 0.36 to 3.24 × 10^−10^, 0.26 to 3.15 × 10^−10^, and 2.34 to 12.99 × 10^−10^ for children, respectively. In general, except for Zn, the noncarcinogenic risks were greater in the reservoir area, in the latter season, and most notably for children. These results may be explained by the trapping effects of the XLD, which causes the accumulation of these HMs in the reservoir area over time (Table 1), as well as by the fact that children are more susceptible to the health risk of HMs than adults [22].

### 3.3. Statistical Analyses for Heavy Metals in Surface Water

**Figure 5:**
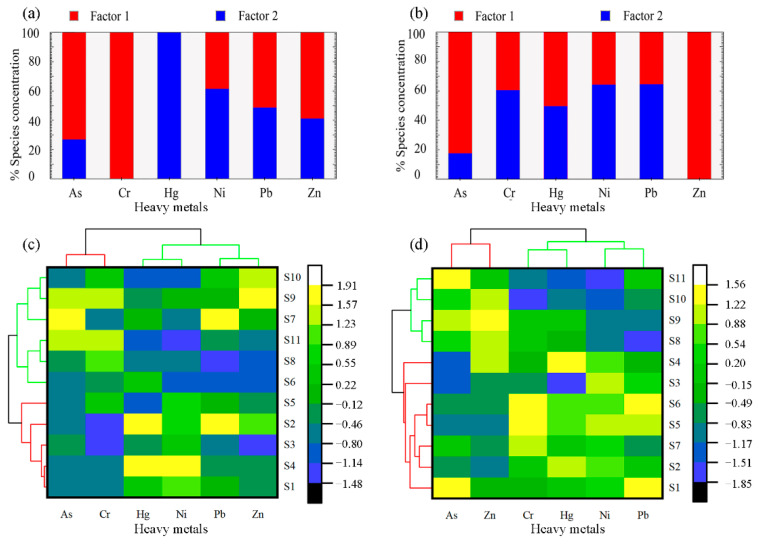
Potential sources of HMs in surface water in the XLD Reservoir and its downstream reach following the WSRS in the post-WSRS I (**a**,**c**) and II (**b**,**d**) seasons, based on PMF (**a**,**b**) and HCA (**c**,**d**).

To better understand the potential sources and related behaviors of HMs in surface water in the seasons following the WSRS, positive matrix factorization (PMF) and hierarchical cluster analysis (HCA) were performed. As shown in Figure 5, the PMF results indicated that, in the former season (Figure 5a), factor 1 contributed 59% of the total HMs in surface water and was mainly dominated by Cr (100%), As (73.2%), and Zn (58.9%); and factor 2 had a degree of interpretation of 27% and was weighted heavily on Hg (99.9%), Ni (61.3%), and Pb (51.3%). In the post-WSRS II season (Figure 5b), factor 1 contributed 50% of the total HMs in surface water and was mainly dominated by Zn (100%), As (82.4%), and Hg (50.4%); and factor 2 had a degree of interpretation of 2% and was weighted heavily on Ni (64.2%), Pb (64.4%), Cr (60.4%), and Hg (49.6%). Comparably, the HCA results were basically in agreement with those of PMF, where all the selected HMs were grouped into two significant categories. In the former season (Figure 5c), the first group included Cr and As, while the second contained Hg, Ni, Pb, and Zn. In the latter season (Figure 5d), the first group included As and Zn, and the second was composed of Hg, Ni, Pb, and Cr.

Combining the results of PMF, HCA, and the spatial and temporal variations in HM concentrations together during the former season, it can be inferred that Cr, As, and Zn likely came from common sources, while Hg, Ni, and Pb originated from another common source. By contrast, during the latter season, As and Zn likely originate from common sources, while Hg, Ni, Pb, and Cr originated from another common source. According to previous studies, the sources of HMs in surface water can be classified as natural and anthropogenic sources [53,54]. In this study, concentrations of Cr, As, and Zn in the downstream reach were higher than those in the reservoir area during the former season (Table 1), indicating that these three HMs may likely be anthropogenic inputs. Previous reports suggest that Cr, As, and Zn mainly originate from industrial activities such as mineral mining and machinery manufacturing [22], while there are other researchers who reported that As and Zn mainly were associated with the abuse of phosphate fertilizer and pesticides [55]. In the downstream reach, the river channels are characterized by the “perched” riverbed surrounded by intensive agricultural activities with almost no industrial activities [21,25]. Therefore, we deduce that Cr, As, and Zn in the former season may be greatly attributed to agricultural activities. However, the higher value of these three HMs may also be explained by the desorption from the deposited sediments during the WSRS [21]. Concentrations of Hg, Ni, and Pb in the reservoir area were higher than those observed in the downstream reach (Table 1), indicating that the above HMs could be attributed to an accumulation from natural origins [22]. The XLD traps fine-grained loess sediments that have eroded from the Chinese Loess Plateau, which, consequentially, causes the accompanied contaminants to accumulate in the reservoir in both the particulate and dissolved forms [12,49]. Therefore, Hg, Ni, and Pb, in the first stage after the WSRS, were likely derived from the natural weathering source upstream from the reservoir.

In the latter stage following the WSRS, As and Zn should also be attributed to common sources of agricultural activities. However, Hg, Ni, Pb, and Cr, which showed significant accumulation in the reservoir during the later stage in comparison with those during the first stage and those in the downstream reach, are likely derived from both natural and anthropogenic sources. This is evidenced by the increased concentration of these four HMs in the downstream reach (Table 1). On one hand, the continuous accumulation of HMs in the reservoir originating from natural sources undoubtedly could lead to increased HM concentrations. On the other hand, the XLD controls approximately 90% of the Yellow River basin, where the anabatic point and non-point discharge in the upper and middle reaches may likely contribute to the increased concentration of these four HMs as well [20]. Thus, the trapping effects of the XLD could serve as anthropogenic and natural sources, leading to the increased concentration of these four HMs [11]. However, it is difficult to accurately determine the contribution rate between natural and anthropogenic sources of these HMs, both in this study and in previous studies [21,22,25,56].

To establish the relationship between sampling sites according to their similarities, HCA was performed on HM concentrations. As shown in the left vertical dendrogram of each heat map, each of the 11 sites was classified into two major groups according to HM concentrations. In the former season (Figure 5c), group 1 was comprised of sites S1–S5, while group 2 comprised of sites S6–S11. In the latter season (Figure 5d), group 1 was comprised of sites S1–S7, and then group 2 comprised of sites S8–S11. The sites in each group displayed similar features and indicated potentially analogous contributing sources [43]. Based on these observations, the distribution of HMs at S1–S5 in the reservoir area should likely have common geochemical behaviors [57], as well as those at S6–S11 in the downstream reach. This can be determined by the variations in hydrological characteristics between the reservoir and the downstream ranch [45]. However, in the latter season, the HM concentrations at sites S6 and S7 downstream of the dam exhibited common geochemical behaviors with S1–S5, which is likely a result of the release of water from the reservoir. Therefore, further analysis regarding the relationship between hydrological characteristics and concentrations of HMs is needed (Section 3.4).

### 3.4. Relationship between Hydrological Characteristics and Concentration of Heavy Metals in Surface Water

**Figure 6:**
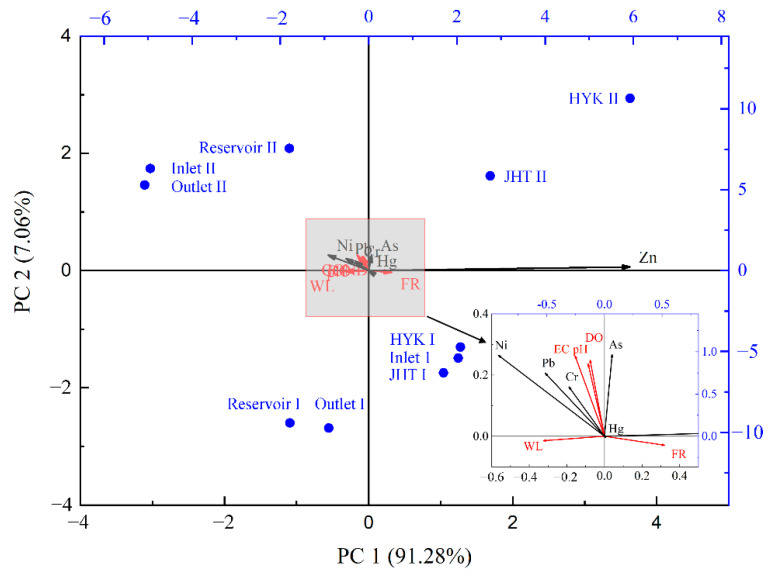
Biplot from redundancy analysis (RDA) showing the relationship between hydrological characteristics and heavy metal concentrations in surface water in the Xiaolangdi Reservoir and its downstream reach. Inlet, Reservoir, Outlet, HYK, and JHT represent sample sites located near the inlet (S1 and S2), reservoir (S3, S4, and S5), outlet (S6 and S7), Huayuankou (S8 and S9), and Jihetan stations (S10 and S11), respectively. I and II represent the post-WSRS I and post-WSRS II seasons, respectively. The environmental variables include water level (WL), flow rate (FR), pH, dissolved oxygen (DO), and electrical conductivity (EC).

Hydrological characteristics play a key role in determining the geochemical behaviors of HMs in aquatic environments [45,57]. In this study, based on the redundancy analysis (RDA), the relationships between hydrological and physicochemical characteristics and HM concentrations in surface water were determined. The hydrological characteristics considered here include water level (WL) and flow rate (FL), while the physicochemical characteristics include pH, dissolved oxygen (DO), and electrical conductivity (EC). The RDA results shown in Figure 6 indicated that the first two explanatory variables explained 98.34% of the total variation between HMs and sample sites. Regarding the sample sites, HM concentrations at the reservoir (S3–S6) and the outlet (S6–S7) stations in the former season were correlated with changes in WL, while those at the inlet of the reservoir (S1–S2) and the downstream stations (HYK and JHT: S8–S11) in the former season were correlated with changes in flow rate. In the latter season, changes in HM concentrations at the inlet (S1–S2), reservoir (S3–S5), and outlet (S6–S7) stations were all correlated with changes in physicochemical variables (pH, DO, and EC). Regarding individual HMs, Zn concentration was positively correlated with FR and was negatively correlated with WL; As concentration was positively correlated with pH, DO, and EC; Ni, Pb, and Zn concentrations were positively correlated with WL, pH, DO, and EC and negatively correlated with FR. In comparison to HMs listed above, the concentration of Hg was less influenced by hydrological characteristics in comparison.

In general, the RDA results indicate that HM concentrations in the former season were mainly influenced by WL and FR, while in the latter season, HM concentrations were mainly influenced by pH, DO, and EC. These results may be attributed to the changes in hydrological characteristics over the different stages following the WSRS [45,57]. During the initial stage following the WSRS, the XLD Reservoir was at the impoundment stage, with a large amount of suspended material. Therefore, the relatively high and unstable fluctuations in water level and flow rate could influence the equilibrium state between the deposition and suspension processes of sediment, simultaneously affecting the adsorption–desorption equilibrium and, ultimately, the transformation of HMs in water [22]. Meanwhile, unstable hydrological characteristics can lead to abnormal hydrodynamics, including the turbulence, flow velocity, and hydraulic residence time during the rising limb of the hydrograph [2,58], which can affect the distribution and migration of HMs in both the reservoir and the downstream reach. In addition to the hydrodynamic conditions, hydrological characteristics play a vital role in causing variations in physicochemical conditions (e.g., pH, DO, and EC) of surface water, as extensively reported in the literature [5,50]. Therefore, as long as the hydrological characteristics remain unstable, variations (i.e., disequilibrium state) in HM concentrations may persist [11]. In contrast, during the latter season, the hydrological characteristics were characterized by a stable water level and flow rate, while variations in surface water HM concentrations were closely related to physicochemical conditions (e.g., pH, DO, and EC) that are determined by certain hydrological and hydraulic characteristics [5,50]. These factors likely explain the low explanatory ability (7.06%) of the second axis, which was dominated by the physicochemical conditions (pH, DO, and EC), and may also inform the high explanatory ability (91.28%) of the first axis, which was dominated by the hydrological characteristics for the RDA results in this study. However, the hydraulic characteristics at each station were not measured and the measured physicochemical characteristics were insufficient in this study. For this reason, further investigations on the effects of hydrological, hydraulic, and physicochemical parameters on HM concentrations in surface water following the WSRS are needed in future work.

## 4. Conclusions

HM concentrations in the surface water of the reservoir and downstream reach of the XLD in the two seasons following the WSRS were well below the drinking water standards in China and those offered by the WHO. However, likely due to the trapping effects of the XLD, concentrations of As, Cr, Hg, Ni, and Pb in both the reservoir and the downstream reach in the post-WSRS II season were significantly higher (*p* < 0.05) than those in the post-WSRS I season. Spatially, concentrations of As in the reservoir were significantly lower (*p* < 0.05) than those in the downstream reach, while concentrations of Ni showed the opposite trend. Meanwhile, HM concentrations increased with decreased distance to the dam in the post-WSRS II season. The contamination of HMs, carcinogenic risks via exposure to As, and noncarcinogenic risks via exposure to Hg, Ni, Pb, and Zn via the direct ingestion pathway of drinking water were all within the acceptable range following the WSRS. However, contamination and health risks of the above HMs increased over time and decreased along flow direction (except for noncarcinogenic risks associated with Zn). Likely due to the trapping effects of the XLD, the carcinogenic risk of Cr intake through the ingestion pathway for adults and children in the post-WSRS II season was at an unacceptably high level, particularly at sites near the dam (S5 and S6). In the post-WSRS I season, Cr, As, and Zn likely originated from agricultural activities, while Hg, Ni, and Pb should be greatly attributed to natural weathering sources upstream from the reservoir. In the post-WSRS II season, As and Zn also likely originated from common agricultural activities, while Hg, Ni, Pb, and Cr were most likely derived from both natural and anthropogenic sources. HM concentrations were mainly affected by hydrological characteristics (WL and FR) in the post-WSRS I season, while HM concentrations were mainly affected by physicochemical parameters (pH, DO, and EC) in the post-WSRS II season. In conclusion, further investigation regarding the effects of hydrological, hydraulic, and physicochemical parameters on HM concentrations in surface water following the WSRS are needed.

## Figures and Tables

**Table 1:** Heavy metal concentrations in surface water in the reservoir and the downstream reach of the Xiaolangdi Dam in the Yellow River. The effects of season, region, and their interaction were conducted based on two-way analysis of variance with a statistical significance level of *p* < 0.05 (highlighted in bold).

Season	Region	Statistic	As (μg/L)	Cr (μg/L)	Hg (μg/L)	Ni (μg/L)	Pb (μg/L)	Zn (μg/L)
Post-WSRS I	Reservoir (*n* = 5)	Mean	0.36	0.87	0.06	3.88	1.16	34.75
		SD	0.18	0.69	0.05	0.73	0.33	10.83
		CV	0.49	0.79	0.77	0.19	0.29	0.31
	Downstream (*n* = 6)	Mean	1.31	2.23	0.02	1.81	1.10	39.06
		SD	1.09	0.96	0.02	0.49	0.42	14.73
		CV	0.84	0.43	0.91	0.27	0.38	0.38
Post-WSRS II	Reservoir (*n* = 5)	Mean	3.60	4.40	0.09	9.43	5.38	25.61
		SD	1.99	1.30	0.04	0.88	1.19	17.81
		CV	0.55	0.30	0.41	0.09	0.22	0.70
	Downstream (*n* = 6)	Mean	5.51	4.07	0.08	5.38	3.83	40.81
		SD	1.35	1.82	0.02	2.53	1.91	20.07
		CV	0.25	0.45	0.30	0.47	0.50	0.49
Season		F	43.883	23.676	8.282	53.315	46.862	0.277
		Sig.	**0.000**	**0.000**	**0.010**	**0.000**	**0.000**	0.605
		Partial η^2^	0.709	0.568	0.315	0.748	0.722	0.015
Region		F	6.469	0.872	3.549	23.993	2.532	1.931
		Sig.	**0.020**	0.363	0.076	**0.000**	0.129	0.182
		Partial η^2^	0.264	0.046	0.165	0.571	0.123	0.097
Season × Region		F	0.754	2.349	0.062	2.937	2.177	0.602
		Sig.	0.397	0.143	0.362	0.130	0.157	0.448
		Partial η^2^	0.040	0.115	0.046	0.123	0.108	0.032

“Bold” represents a statistical significance level of *p* < 0.05.

## Data Availability

The data presented in this study are available on request from the corresponding author.

## Supplementary Materials

The following supporting information can be downloaded at: https://www.mdpi.com/article/10.3390/ijerph19095713/s1, Table S1: Sampling sites characteristics in the Xiaolangdi Reservoir and the downstream reach in the Yellow River; Table S2: Values of water pollution indices for individual sampling locations in the Xiaolangdi Reservoir and its downstream reach in the middle and lower reaches of the Yellow River, China. Table S3: Standard permissible limit, assigned weights and calculated unit weights of individual heavy metals for the heavy metal contamination index (HCI).
