# Supplementary material for: Concentration, Health Risk, and Hydrological Forcing of Heavy Metals in Surface Water Following Water-Sediment Regulation of the Xiaolangdi Dam in the Yellow River
Source: Int J Environ Res Public Health. 2022 May 7;19(9):5713. doi: 10.3390/ijerph19095713 (PMC9099827; doi:10.3390/ijerph19095713)
Supplement: Supplementary file 1 [file ijerph-19-05713-s001.zip › ijerph-1688517-supplementary.pdf]

Supplementary Materials

Concentration, Health Risk, and Hydrological Forcing of Heavy Metals in Surface Water Following Water-Sediment Regulation of the Xiaolangdi Dam in the Yellow River

Qinghe Zhao, Shengyan Ding *, Zihan Geng, Zhendong Hong, Jinhai Yu, Yi Liu

Key Laboratory of Geospatial Technology for the Middle and Lower Yellow River Regions of the Ministry of Education, College of Geography and Environmental Science, Henan University, Kaifeng, Henan 475004, China

* Corresponding author (Shengyan Ding), e-mail address: syding@henu.edu.cn

Table S1. Sampling sites characteristics in the Xiaolangdi Reservoir and the downstream reach in the Yellow River.

No.	Region	Longitude (°)	Latitude (°)	Elevation (m)	Distance to dam (km)
S1	Reservoir	E111.356	N34.821	272.4	126.3
S2	Reservoir	E111.601	N34.892	253.9	99.3
S3	Reservoir	E111.810	N35.055	255.6	67.4
S4	Reservoir	E112.009	N35.046	246.5	48.6
S5	Reservoir	E112.358	N34.941	251.2	5.9
S6	Downstream	E112.632	N34.850	115.0	23.4
S7	Downstream	E113.077	N34.844	106.1	71.9
S8	Downstream	E113.697	N34.910	91.4	151.4
S9	Downstream	E114.139	N34.958	79.6	201.2
S10	Downstream	E114.496	N34.920	75.3	238.4
S11	Downstream	E114.694	N34.912	74.0	263.4

Table S2. Values of water pollution indices for individual sampling locations in the Xiaolangdoi Reservoir and its downstream reach in the middle and lower reaches of the Yellow River, China.

Season	Region	No.	HEI	Mq-HPI		Mqw-HPI	
				PI	NI	PI	NI
Post-WSRS I	Reservoir	S1	0.25	0	-0.95	0	-0.96
		S2	0.29	0	-0.94	0	-0.96
		S3	0.21	0	-0.96	0	-0.96
		S4	0.24	0	-0.96	0	-0.96
		S5	0.25	0	-0.96	0	-0.96
	Downstream	S6	0.17	0	-0.97	0	-0.97
		S7	0.49	0	-0.89	0	-0.93
		S8	0.20	0	-0.97	0	-0.96
		S9	0.46	0	-0.91	0	-0.93
		S10	0.24	0	-0.96	0	-0.96
		S11	0.42	0	-0.91	0	-0.93
Post-WSRS II	Reservoir	S1	1.59	0	-0.64	0	-0.80
		S2	1.10	0	-0.77	0	-0.86
		S3	0.98	0	-0.80	0	-0.88
		S4	0.83	0	-0.84	0	-0.89
		S5	1.23	0	-0.75	0	-0.85
	Downstream	S6	1.36	0	-0.71	0	-0.84
		S7	1.05	0	-0.78	0	-0.85
		S8	0.84	0	-0.82	0	-0.87
		S9	1.16	0	-0.74	0	-0.83
		S10	0.99	0	-0.77	0	-0.86
		S11	1.31	0	-0.69	0	-0.83

Note: HEI indicates the heavy metal evaluation index; Mq-HPI indicates the modified heavy metal pollution index based on sub index Q; Mqw-HPI indicates the modified heavy metal pollution index based on weight and sub index Q.

Table S3. Standard permissible limit, assigned weights and calculated unit weights of individual heavy metals for the heavy metal contamination index (HCI).

Heavy metal	S_i	Aw_i	W_i
As	10	3	0.200
Cr	50	2	0.133
Hg	6	4	0.267
Ni	70	2	0.133
Pb	10	3	0.200
Zn	3000	1	0.067

Note: Aw_i is the assigned weight for the ith heavy metal based on Delphi's method, and the sum of Aw_i = 15; W_i is the calculated unit weights for the ith heavy metal, and the sum of $W_i = 1$; S_i represents the standard permissible limit for the ith heavy metal in µg/L (World Health Organization) (Mohebbi et al., 2013; Rajkumar et al., 2020).
